# Modelling the lymphatic system: challenges and opportunities

**DOI:** 10.1098/rsif.2011.0751

**Published:** 2012-01-11

**Authors:** K. N. Margaris, R. A. Black

**Affiliations:** Department of Bioengineering, University of Strathclyde, 106 Rottenrow, Glasgow G4 0NW, UK

**Keywords:** lymphatic system, mathematical modelling, biomechanics, physiological flows, computational fluid dynamics

## Abstract

The lymphatic system is a vital part of the circulatory and immune systems, and plays an important role in homeostasis by controlling extracellular fluid volume and in combating infection. Nevertheless, there is a notable disparity in terms of research effort expended in relation to the treatment of lymphatic diseases in contrast to the cardiovascular system. While similarities to the cardiovascular system exist, there are considerable differences in their anatomy and physiology. This review outlines some of the challenges and opportunities for those engaged in modelling biological systems. The study of the lymphatic system is still in its infancy, the vast majority of the models presented in the literature to date having been developed since 2003. The number of distinct models and their variants are few in number, and only one effort has been made thus far to study the entire lymphatic network; elements of the lymphatic system such as the nodes, which act as pumps and reservoirs, have not been addressed by mathematical models. Clearly, more work will be necessary in combination with experimental verification in order to progress and update the knowledge on the function of the lymphatic system. As our knowledge and understanding of its function increase, new and more effective treatments of lymphatic diseases are bound to emerge.

## 1. Introduction

The lymphatic system constitutes a one-way transport system that operates in conjunction with the circulatory system. Its primary function is to transport excess interstitial fluid, from the interstitial space, back to the blood circulation, via the thoracic duct [1]. Along with the excess interstitial fluid, excessive proteins and waste are transported back to the circulation. The lymphatic system also acts as a conduit for immune cells and facilitates the immune response [2]. Lymph nodes across the network filter the interstitial flow and break down bacteria, viruses and waste. The lymphatic system is, therefore, not part of the circulatory system alone; rather, it is an integral part of the immune system. It plays an important role in the dissemination of cancer [3,4]. Its role in transplantation is also significant. Latest findings suggest that early lymphangiogenesis in a transplanted graft may be responsible for early rejection, but it is beneficial later on [5].

Failure of lymph drainage can be a consequence of infection, trauma, surgery, transplantation, medication or venous disease, or it may be congenital [3,6]. The build-up of interstitial fluid results in swelling and pain, and increases the risk of infection. This condition is known as lymphoedema and current treatments have limited success. Arm lymphoedema is very common in breast cancer patients and lower limb lymphoedema may lead to incapacity in severe cases.

Despite the importance of the lymphatic system in health and disease, it remains overlooked in terms of research, especially compared with the circulatory system [7,8]. Although tissue engineering has made some progress towards therapy in other areas, in the lymphatic system it is still in its infancy [4].

The lymphatic system is a highly complex system with highly variable structure and function between anatomical sites and between species. The lack of mathematical models developed for the study of its function presents a wealth of opportunities for researchers. Unfortunately, this is hindered by the lack of anatomical and physiological data.

The present review attempts to summarize the current knowledge on the lymphatic system with an emphasis on the modelling of its biomechanical behaviour. Sections 2 and 3 give a brief overview of the anatomy and physiology of the lymphatic system, respectively. Section 4 deals with the mathematical models developed thus far and discusses their key assumptions and limitations. Finally, §5 anticipates future research.

## 2. Anatomy of the lymphatic system

**Figure 1.**
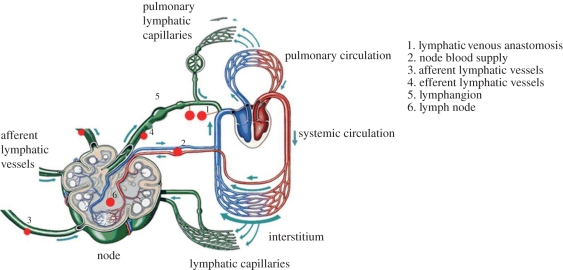
The lymphatic system. Adapted with permission from Quere [9].

The lymphatic system is composed of a network of vessels, termed lymphatics, lymph nodes and lymphoid organs. The interstitial fluid enters through the small lymphatic capillaries (also called initial or terminal lymphatics) that gradually combine to form larger diameter vessels, namely the pre-collectors, collectors, trunks and ducts. The interstitial fluid, which is termed lymph when inside the lymphatics, is pumped slowly by the contraction of the lymphatic vessels, which contain smooth muscle in their walls. Retrograde flow is prevented by a series of one-way valves. Figure 1 shows a simplified schematic diagram of the lymphatic system.

The lymphatic network is asymmetrical: the right-hand side of the head and thorax and the right arm drain into the right subclavian vein, whereas the lymphatic vessels of the rest of the body converge at the thoracic duct, which empties at the junction of the jugular and left subclavian veins.

### 2.1. Initial lymphatics

Initial lymphatic capillaries have a diameter of approximately 10–60 µm with a wall thickness ranging from 50 to 100 nm and are blind-ended. Electron microscopy revealed that lymphatic capillaries are usually collapsed with irregular walls and do not have a continuous basal lamina. They comprise a monolayer of non-fenestrated endothelial cells [4]. They are supported by anchoring filaments (6–10 nm in diameter) that keep them from completely collapsing when interstitial fluid pressure increases [10]. Their unique structure allows them to function as a one-way valve system that allows fluid to flow into them and then to close in order to stop back-flow as the internal pressure rises. Trzewik *et al.* [11] demonstrated this function experimentally, although they were not the first to postulate the existence of primary lymphatic valves. Leak & Burke [10] investigated the structure of the lymphatic capillaries, and speculated that they could function as valves, although at that time no proof was provided.

### 2.2. Pre-collecting lymphatics

The pre-collecting lymphatics connect the capillaries to the collecting vessels. They contain bicuspid one-way valves, but unlike the collecting vessels, where they are located at regular intervals, their distribution becomes more irregular towards the capillary vessels, and may comprise a single leaflet [6,12]. The pre-collectors contain one or more layers of smooth muscle cells within their walls, and are capable of performing spontaneous contractions. However, portions without muscle exist; in these parts, the endothelial layer in the pre-collectors is similar to that of initial lymphatics with a discontinuous basal lamina. Thus, these structures absorb fluid instead and therefore the pre-collectors have a dual role: absorption and propulsion of lymph [12].

### 2.3. Collecting lymphatics

**Figure 2.**
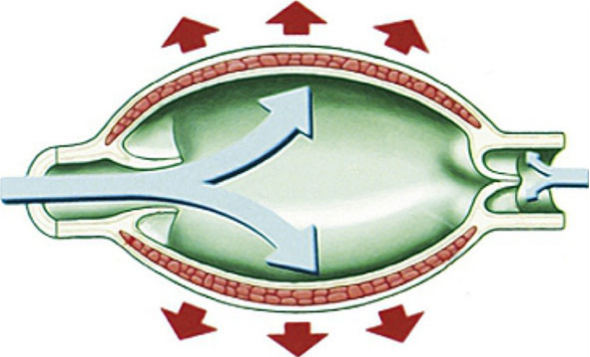
Lymphangion. This schematic diagram cannot encompass the wide variation of vessel aspect ratio observed at different anatomical sites; and while the distension shown in the figure is perhaps exaggerated, lymphangions have been reported to double or triple their diameter during diastole [15,16]. Reproduced with permission from Quere [9].

The larger collecting lymphatics differ from the capillaries and pre-collectors, as they have a complete basal lamina and therefore the primary lymphatic valves are absent. They contain another type of valve that prevents retrograde flow: the secondary valve. The wall structure is similar to that of blood vessels. Three layers can be identified—the intima, media and adventitia [13].^1^ They are composed of endothelial cells, smooth muscle and collagen fibres, respectively. It has been observed that the muscle bundles are arranged in a hellicoidal manner in both collecting and pre-collecting vessels [12]. The part of the vessel between two valves is known as a lymphangion (figure 2).

**Figure 3.**
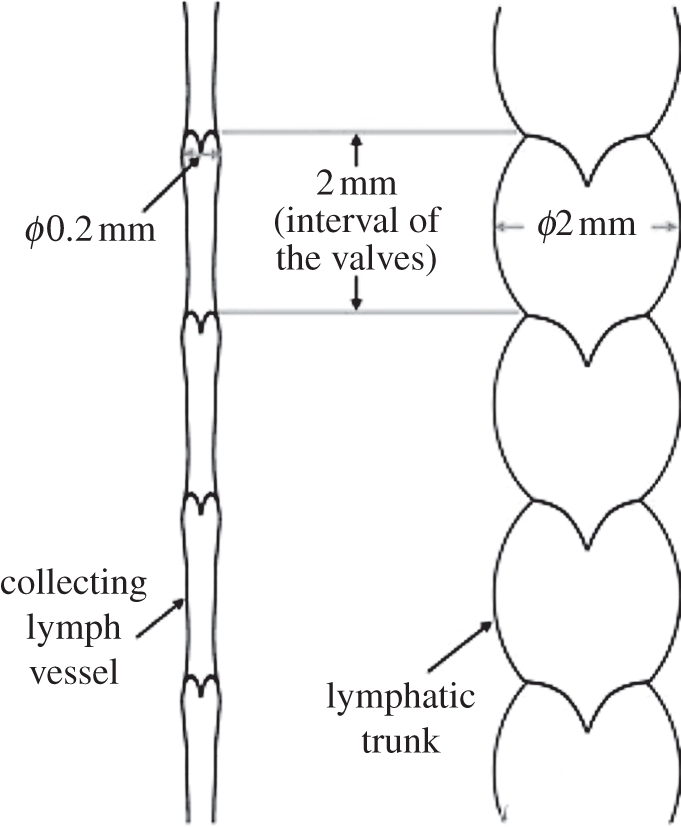
Schematic diagram of a collecting vessel and a trunk/duct of the human head and neck from a cadaveric study. It appears that, at this anatomical site in humans, the intervalvular distance is roughly the same. However, this is not a general case [18]. Reproduced with permission from Pan *et al.* [17].

Lymphangions are innervated with sympathetic and parasympathetic nerves, and can perform rhythmic contractions. In humans, the lymphangions present within the head and neck have an average diameter of 0.2 mm and a length of 2 mm, yielding a diameter to length ratio of 0.1 [17]. Collecting lymphatics are approximately 1–2 mm in diameter in human lower extremities [18]. The largest (and deeper) lymphatic vessels, also referred to as trunks and ducts, have a diameter of the order of 2 mm, and the diameter to length ratio can be close to unity (figure 3). This may have implications for the modelling of the lymph flow, which will be discussed in §2.4.

### 2.4. Secondary valves

Generally, the lymphatic secondary valves are of the bicuspid type, regardless of species studied [19,20]. However, up to five leaflet valves as well as single leaflet ones have been observed [21]. Data regarding the size, the geometry and the number of valves are scarce, and, in humans especially, there is a lack of data on the structure and operation of the lymphatic valves [22], although valve size varies with the vessel calibre [17].

Studies of the rat spinotrapezius muscle by Mazzoni *et al.* [20] have shown that smooth muscle cells are absent from the valve leaflets, indicating that these are passive structures.^2^ The valves comprise a monolayer of endothelial cells on a collagen matrix. Mazzoni *et al.* [20] suggested that the valve operation is determined by the pressure and viscous forces associated with the lymph flow. Based on the anatomy of valves studied and the presence of a buttress structure, the authors speculated that tension from the surrounding tissues may also contribute to the valve function. Recently, Davis *et al.* [23] investigated the valve gating in rat mesenteric lymphatics. It was observed that opening and closing of valves were occurring only because of a pressure gradient; however, the vessel muscle tone influenced the required pressure gradient. Studies of the thoracic duct of monkeys by Lee *et al.* [19] have shown the presence of bicuspid valves, but no buttress structure, indicating that there may be differences in the details of valve operation depending on species and anatomical site.

### 2.5. Lymphoid organs

Lymphoid organs are classified as being either primary or secondary organs. The primary organs are the thymus and bone marrow, and are responsible for the production and maturation of lymphocytes. The secondary organs include the spleen, Peyer's patches, appendix, the tonsils and the nodes. They are responsible for further maturation of lymphocytes and initiation of an immune response. Only the structure and function of the lymph nodes will be discussed in the context of this paper, as they are the only lymphoid organs that play a role in the active transport of lymph [9].

**Figure 4.**
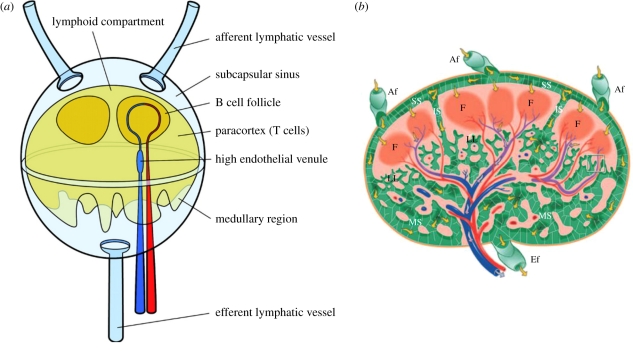
(*a*) Schematic diagram of a lymph node. Lymph flows around the lymphoid compartment and part of it enters the paracortex and follicles, where cells of the immune system remove pathogens and foreign material. Reproduced with permission from Roozendaal *et al.* [25]. (*b*) Illustration of a node cross section. Red and blue denote arteries and veins, respectively. Yellow arrows indicate lymph flow. SS, subcapsular sinuses; LL, lymphatic labyrinth; F, follicle; MS, medullary sinuses; IS, intermediate sinuses; Af, afferent; Ef, efferent. Reproduced with permission from Ohtani & Ohtani [24].

The structure of the node is highly complex and not easily visualized, owing to the large number of cells that are resident within it [24]. A representation of the lymph node structure is shown in figure 4*a*, and a more detailed cross section is shown in figure 4*b*.

The exterior walls of the node are covered with lymphatic smooth muscle, thus exhibiting contractile behaviour like the lymphatic vessels, although the frequency of contraction is lower. Hughes & Allen [26] and McGeown & Gallagher [27] report a frequency of 0.78 b.p.m. in bovine nodes, while Thornbury *et al.* [28] observed contractions at a frequency of 0.5–1 b.p.m. in ovine nodes. Tumer *et al.* [29] performed a frequency analysis and found the dominant rhythms to lie within the ranges of 0.01–0.04, 0.05–0.07 and 0.09–0.14 Hz. In contrast, lymphatic collecting vessels contract at a frequency in the range of 0.6–10 b.p.m. [30–33], although up to 30 b.p.m. have been reported in the literature [34]. Limited information exists on human nodal contractility, its presence and its regulating mechanisms [22].

Lymph nodes present a relatively high resistance to flow, which depends on the flow rate of lymph [35,36]. Papp *et al.* [36] reported that the resistance of the node in dogs was 100 times larger than that of the thoracic duct, while Browse *et al.* [35] found that the resistance of the nodes fell by up to six times as the flow rate in the afferent lymphatics increases. They concluded that the reduction in resistance was due to swelling of the nodes, which created more space for lymph to flow, thereby reducing their resistance. Nevertheless, despite this reduction, the nodes still impose a large resistance to the flow.

It is not surprising that the nodes have a large resistance, if one considers their internal structure. Lymph flows into the subcapsular sinus and is immediately forced to flow around the lymphoid compartment^3^ and into a network of sinuses (yellow arrows in figure 4*b*), where it is filtered by macrophages that reside on the outer surface of the lymphoid compartment. Most of the lymph exits the node from the efferent lymphatics without entering the lymphoid compartment. While in figure 4 multiple afferents and one efferent vessel are shown, any possible combination of the number of these vessels has been observed in humans [17] as well as in mice [38]. Kowala & Schoefl [38] also observed that there is no constant relationship in the number of afferent vessels and the number of nodal compartments. Whether this variation in numbers of vessels is for overall node resistance reduction, increased lymph-filtering efficiency or both is uncertain.

The outer surface of the lymphoid compartment is covered by sinus-lining cells that create an almost impermeable membrane [25]. Large molecules cannot penetrate this layer, thus creating an effective barrier that prevents pathogens from reaching the bloodstream. Lymphocytes, lymph and smaller molecules can enter through specialized channels [24,25]. The exclusion of large molecules from the lymphoid compartment explains the well-established fact that the protein composition of efferent lymph is higher than that of the afferent one, although the exact mechanisms of mass exchange are not fully understood.

The amount of lymph entering is low and therefore the inside of the lymphoid compartment is relatively fluid-free, although controversy in the literature does exist as, according to Tretbar *et al.* [6], up to 50 per cent of the lymph is lost inside this compartment. Lymph flows within specialized channels, referred to as the conduit system [25,39], that start from the sinus-lining cells and end on the lumen of venules that penetrate inside the lymphoid compartment. The cells lining these venules are specially adapted to allow lymphocytes to freely enter the node from the blood and are named high endothelial venules (HEVs). HEVs also allow lymph to return to the bloodstream.

## 3. Physiology of the lymphatic system

The interstitial fluid is taken up by the small lymphatic capillaries in a process known as lymph formation. Lymph is then pumped against a pressure gradient towards the jugular vein, by rhythmic contractions of the lymphangions and by external motion of skeletal muscle, arteries and veins. This process is referred to as lymph propulsion. Lymph formation depends on external (or extrinsic), but local, effects such as interstitial fluid pressure and extracellular matrix strain and is thus a local process [3]. On the other hand, lymph propulsion is a systemic process regulated by a large number of factors: streamwise pressure gradients, transmural pressure, nerves and hormones.

### 3.1. Lymph formation

Several mechanisms have been proposed for the formation of lymph in initial lymphatics. Of those, only the interstitial pressure and volume theory have been widely accepted [40]. Yet, the exact regulatory mechanisms are unclear [41].

Lymph enters through openings in the endothelial layer of the capillaries, which act as one-way valves. They open when interstitial pressure increases and permit interstitial fluid flow inside the initial lymphatics. As the pressure inside the vessel rises, the valve closes. Flow inside the initial lymphatics is thought to be facilitated by fluctuations in interstitial fluid pressure and by the suction of the collecting lymphatics downstream [42]. Studies in a number of mammalian species (rodents, canines, ovines, bovines, humans) have shown no evidence of smooth muscle cells in the initial lymphatics. It follows that the driving force of lymph formation must be the result of extrinsic factors. The sole exception to this is the bat [34,40].

Lymph formation is organ dependent because of the differences in the structure and mechanical properties between tissues [43].

### 3.2. Lymph propulsion

After the filling of the initial lymphatics, lymph has to be pumped against a pressure gradient. Lymph propulsion is performed by the rhythmic contractions of the smooth muscles of lymphangions and facilitated by the presence of the one-way valves that prevent retrograde flow. The contractions propagate from one lymphangion to the next like a wave that causes both radial contraction and axial shortening of the vessels [33,44]. It has been reported that lymphangions rotate *in vitro* as well [45], most probably owing to the helical disposition of these cells within the vessel wall [12]. Although there is evidence to support that the contraction wave is transmitted between lymphangions through endothelial gap junctions [33], it has been observed that the propagation of the wave may become discontinuous. This suggests that lymphangions have a self-regulating mechanism mediated by pacemaker cells that are thought to reside downstream of the valves [31]. The contraction wave can travel in an orthograde or retrograde direction. Evidence shows that either propagation direction is equally likely to take place [33] and the volume of fluid pumped is not significantly affected by it [31].

However, there is some controversy regarding the nature of individual lymphangion contraction. The accepted view is that lymphangions contract in a peristaltic manner, that is, the contraction is a radial constriction of the vessel travelling along its length, similar to the oesophagus or the ureter [3,21]. Evidence supporting this view exists in the literature [32], with 84 per cent of the contractions observed propagating along the entire length of the lymphangion [33]. Peristalsis in guinea pig mesenteric lymphatics has also been reported [44]. The peristaltic character of contraction is disputed by others (J. E. Moore 2011, personal communication), and videos captured with a high-speed camera by Davis *et al.* [23] show the entire wall contracting at roughly the same time. It should be noted that these studies were conducted in different species: bovine in Ohhashi *et al.* [32] and rats in Davis *et al.* [23].

Lymph propulsion in collecting vessels is affected by preload, afterload, transmural pressure and shear stresses in addition to nerve and humoral mediators. These are termed intrinsic mechanisms. The details of these mechanisms and, especially, the molecular regulation are not fully understood [46]. Extrinsic mechanisms such as skeletal muscle contraction, motion of adjacent organs and arterial pulsations also influence lymph flow. External forces such as massaging, a common treatment modality for lymphoedema, were once thought to affect lymph flow but evidence shows that their effect is on the filling of initial lymphatics rather than on the pumping of the larger collecting vessels [47].

It is not clear whether extrinsic mechanisms can have a significant or even dominant role in the pumping of lymph. Olszewski [48] and Olszewski & Engeset [49] consider extrinsic mechanisms to be secondary to the intrinsic pumping based on their results in humans. On the other hand, Negrini *et al.* [50] found that, in rabbits, pleural lymph flow is due to respiratory movements of up to 40 per cent of the total volume, although it cannot be excluded that, at other sites, the intrinsic mechanism is not the dominant one.

Passive flow owing to a positive pressure gradient may also occur in oedema, during which lymph formation is increased [47]. In fact, Gashev *et al.* [51] report that, for low levels of lymph formation, the intrinsic mechanism is dominant but, as these levels rise, the active lymph pump is inhibited and the vessels become conduits. However, according to the authors, this does not prove that the flow-related inhibition decreases total flow *in vivo*, since, at higher levels of lymph formation, passive flow may be dominant.

The lymphatic structure and function are very complicated and can only be briefly addressed in the context of the present review. The reader is referred to the reviews by Aukland & Reed [40], Aukland [47], Gashev & Zawieja [22,41], Reddy [52], Schmid-Schonbein [34,43] and Swartz [3], the textbook by Földi *et al.* [7] and the references therein for more information. Unfortunately, the information on the human lymphatic system is scarce. Most studies have been performed in different animal species with different experimental procedures at different locations, which adds to the confusion. Extrapolation of results to humans should be performed with care as there are differences in species as well as differences between members of the same species. It is not surprising therefore that there are still structures in lymphatic vessels found in humans (ampullae and diverticula) for which no explanation of their function exists [17].

## 4. Mathematical modelling

Mathematical modelling of the lymphatic system is in its infancy. The first model of the entire lymphatic system is attributed to Reddy [53]. It is a simplified one-dimensional model of the entire lymphatic network that is based on the Navier–Stokes equations of fluid mechanics. Although parts of the lymphatic anatomy had to be excluded for simplicity or because of a lack of available information, this was a pioneering work at that time and remains the only model of the entire lymphatic circulation. In contrast, the arterial tree has attracted more attention, with upwards of 23 models cited in the literature to date [54].

Recently, the lymphatic system has attracted more attention. Macdonald *et al.* [45] refined the lymphangion model proposed by Reddy *et al.* [55], although they modelled only a short chain of lymphangions. Bertram *et al.* [56] modelled a chain of lymphangions as well, using a lumped approach, with valve resistance varying with the pressure gradient across the valve. Other researchers have used similar lumped approaches. Quick *et al.* [57] and Venugopal *et al.* [58] used a simplified algebraic equation based on the time-varying elastance concept employed in cardiovascular dynamics. Drake *et al.* [59] employed an equivalent circuit technique.

The models mentioned above are either lumped or one-dimensional. There are even fewer models in higher dimensions. In fact, to the authors' knowledge, only one three-dimensional model of a single lymphangion, albeit neglecting the valves, has been published so far by Rahbar & Moore [60].

A few models of the initial lymphatics have been developed as well. The first attempt is arguably that of Reddy & Patel [42]. Mendoza & Schmid-Schonbein [61] developed a two-dimensional model of the primary lymphatic valves, which was later refined by Galie & Spilker [62]. Recently, Roose & Swartz [63] developed a multi-scale model of the initial lymphatics using homogenization theory in order to study the optimal structure of the lymphatic capillary network with respect to interstitial fluid drainage.

Models of the secondary valves are currently lacking. Macdonald [64] included a very simple two-dimensional model of a secondary lymphatic valve, although this was a very crude model in which the motion of the valve leaflet was imposed rather than solved through a fluid–structure interaction model.

The models that exist so far in the literature can be classified into two categories: lumped and continuum models. In the context of the present text, a continuum model refers to a model described by partial differential equations as opposed to lumped models that are described by ordinary differential or algebraic equations. In §§4.1 and 4.2, the abovementioned models are discussed and their limitations are elucidated.

### 4.1. Lumped models

One type of approach that can be used to model lymph flow is the lumped approach in which a distributed system is reduced to a discrete one. Obviously, these models are simple and the mathematical complications are kept to a minimum. They can yield useful insight into the behaviour of the system under investigation; however, they cannot yield a detailed description of the flow.

Drake *et al.* [59] used an electrical analogue model of lymphatic vessels. The vessel was modelled as a resistance and a voltage source to take into account the flow resistance and pumping action of the vessel, respectively. A diode was added to account for the valves. This very simple approach allows the inclusion of a large number of vessels, or regions of the lymphatic circulation, by lumping them into an equivalent resistance and voltage and connecting the resulting equivalent series in series or parallel. However, the authors used the Thevenin theorem in their analysis, which imposes the requirement that the pressure–flow rate relationship is linear. Depending on the species or lymphatic compartment under consideration, this relationship is, in general, highly nonlinear [32,65–67]. Thus, the applicability of such models is questionable.

The same group undertook additional work using a model composed of a linear circuit component and a nonlinear model of a lymphatic pump composed of five lymphangions. Both passive and active flow were taken into account and, despite its simplicity, the model showed that, when outflow pressure is higher than inflow pressure, flow is mainly due to the active contractions, but the pressure gradient becomes positive when most of the flow is due to passive factors [65,68]. This is in line with the experimental findings by Gashev *et al.* [51].

Quick *et al.* [57] developed a lumped model by employing the time-varying elastance approach, which was originally developed for the ventricles of the heart [69]. This model was compared with a more complex lumped model, derived by Quick and co-workers [70,71] using the linearized Navier–Stokes equations of fluid flow. Quick *et al.* [57] claimed that the simplification of the their model to a simple algebraic equation, by neglecting inertia and the viscosity effect, is justifiable and the error introduced in mean flow is small. However, in the vicinity of the valves, the inertial terms are expected to be dominant, according to Reddy & Kesavan [72].

Venugopal *et al.* [58] refined the time-varying elastance model using a bilinear approximation for the pressure–volume relationship, which is certainly an improvement as the actual P–V relationship is nonlinear and has been approximated with an exponential function by Ohhashi *et al.* [32]. The hypothesis proposed is that lymphangions change behaviour depending on the magnitude of the transmural pressure: at higher transmural pressures, lymphangions become insensitive to it and maintain constant stroke volume.

Bertram *et al.* [56] modelled a chain of lymphangions and concentrated on the pumping behaviour and derived pump-characteristic curves. As with the rest of the lumped models discussed in this section, a peristaltic wave of contraction cannot be modelled without the addition of more lumped components. This model added a pressure-dependent valve resistance, and is the first modelling attempt to do so. The constitutive equation of the vessel wall included passive elasticity and active contraction terms. The inclusion of these terms allowed for wall stiffening at high internal pressures and loss of compliance at high external ones. The resulting nonlinear ordinary differential equations yielded a complicated behaviour, which depended on the parameters and the number of lymphangions in a chain.

An interesting result of this model was that coordinated pumping between lymphangions is inefficient, which contradicts the results derived from the time-varying elastance model by Venugopal *et al.* [71]. The contraction waves have been observed *in vitro* to propagate downstream or upstream with a finite velocity of 5–8 mm s^−1^. *In vivo* contractions can be irregular and lymphangions have a refractory period, which is not included in the work by Bertram *et al.* [56]. Lymphangions do not contract synchronously and consequently the result by Bertram *et al.* [56] is likely to be more realistic, even with the aforementioned limitations, than the ones obtained from the time-varying elastance models. In the work by Venugopal *et al.* [71], an experimental verification was also provided, but the experiments were conducted in valveless lymphangions. Therefore, the results or conclusions from this study must be interpreted with caution, owing to the importance of the one-way valve system in lymphatic flow generation. It was also found that pumping is less effective as the lymphangion diameter decreases owing to an increase in external pressure, indicating that when oedema is present collecting vessels in the affected region will not be able to contribute to lymph clearance by active contraction.

While simple approaches such as the work by Quick *et al.* [57] and Venugopal *et al.* [71] discussed in this section are useful, and can provide useful insight into the function of the lymphatic system when used with caution, their limitations should not be overlooked. The elastance is sensitive to pressure changes, and the nonlinear pressure–volume behaviour of lymphangions limits the applicability of this concept to the study of the lymphatic system [67]. Moreover, there are differences in the behaviour of lymphangions and ventricles [58] and the time-varying elastance model has been criticized as insufficient to describe the ventricle dynamics as well, for which it was initially developed [73,74].

Reddy [53] was the first to develop a model of a large part of the lymphatic network. The model is derived from the one-dimensional Navier–Stokes equations [72,75], by assuming that inertial effects are negligible. The lymphatic vessels were assumed to be contracting, although a simplification was made by assuming that the vessels have a uniform radius over the length, i.e. they retain a cylindrical shape. Spatial averages of the pressure were used for each lymphangion, and by doing so the Navier–Stokes equations reduced to ordinary differential equations, and hence this model is effectively a lumped one.

After deriving the equations describing fluid flow and momentum transport of a single lymphangion, Reddy and co-workers [55,75] proceeded to assemble them into a model of the thoracic duct, or a network model of the whole system, although several simplifications were necessary. The initial lymphatics were considered to be lumped. The nodes were not considered owing to the fact that information on the microcirculation inside them was not available, although it is thought that they affect lymph flow owing to their contractility, high resistance and mass exchange processes that take place inside them. The model was completed via the incorporation of the following boundary conditions: pressure in the jugular vein, at arterial and venous capillaries, capillary protein concentration and external pressure exerted on each lymphangion. It should be noted that this is the only model that takes into account the refractory period of lymphangions. The results, according to these authors, were in agreement with experimental observations in sheep by Hall *et al.* [76] when extrapolated in humans. However, the validity of this approach is questioned by Olszewski & Engeset [49].

There are several aspects of lymphangion behaviour that have not been addressed by lumped models; in fact, they are not addressed by continuum models either. These aspects are the dependence of contraction on transmural pressure and shear stresses, the influence of humoral factors and nerve stimulation.

Lumped models can nevertheless yield a very complicated behaviour, as shown by Reddy [53] and Bertram *et al.* [56], but cannot resolve space-dependent flow characteristics, at least without the inclusion of more lumped components. This is where more advanced computational models can have an advantage.

### 4.2. Continuum models

Continuum models of the lymphatic system are scarce. As mentioned previously, Macdonald *et al.* [45] refined Reddy's mathematical model by including two terms in the transmural pressure equation: a tension term and a damping term. Moreover, spatial averages of the pressure were not assumed, thus retaining the continuum nature of the governing equations. Two notable differences are that the contraction of the vessel wall was modelled by a change in Young's modulus rather than an active stress added to the stress state of the material, and it was assumed that lymphangions do not have a refractory period, as in the work by Reddy *et al.* [75]. Data for parameters such as the Young modulus and damping were derived by experiments on bovine mesenteric lymphatics, although the properties measured had a very large variation owing to uncertainties in vessel diameter and wall thickness measurements.^4^ The Young modulus was found to be 1.2 ± 0.7 kPa; a quite large variation, possibly even larger than the physiological variations that one would expect to find between individual members of species. The damping term also varied considerably.

The models discussed so far, either lumped or continuum, have some common limitations. First of all, they assume one-dimensional laminar flow with a parabolic velocity profile (i.e. fully developed), whereas in reality, based on anatomical findings, the length to diameter ratio varies widely depending on the anatomical site. While the laminar flow assumption may be valid for lengthy vessels, this is not necessarily the case for shorter vessels with valves at either end, where the flow field is likely to be substantially different. Reynolds numbers of up to 5 have been reported in the literature [77], and while these values are low it must be emphasized that, if the values reported are correct, the inertia forces are by definition five times greater than viscous ones, giving rise to the possibility of localized eddy formation [44] and areas of recirculation and mixing. (Even in the case of a Reynolds number of 1, the inertia and viscous forces are of equal magnitude and their effect should be studied further before concluding that they can be neglected.)

Further complications may arise when the flow is unsteady, and the classical definition of Reynolds number, *Re* = *ρud*/*μ*, which has been used in the literature to characterize lymph flow is not appropriate in this case. In peristaltic flows, the modified Reynolds number quoted by Shapiro *et al.* [78] is more appropriate: *Re* = *ρα*^2^*c*/*μλ*, where *c* is the wave propagation velocity, *α* is the mean half-width of the channel (mean radius in cylindrical channels) and *λ* is the wavelength.

While the formation of turbulence in the vicinity of the valves is possible according to Gashev [79], evidence for the existence of turbulence in lymphatics is not found elsewhere in the literature. Studies of flow within microfluidic devices show that transition to turbulent flow depends on the entrance length, and may take place at Reynolds numbers far lower than that of fully developed pipe flows [80]. Nevertheless, true turbulence is a phenomenon that needs time and space to develop, and the evidence available to us at this time suggests that its formation within the lymphatic system is highly unlikely.

Recently, Rahbar & Moore [60] challenged the assumption of one-dimensional flow by developing a three-dimensional computational model of a contracting lymphangion, without valves and inertial effects, and still assuming a slender vessel with a maximum aspect ratio of 3.75. This is not a true fluid–structure interaction simulation, though, as the wall motion was prescribed as a boundary condition.

Comparison of the wall shear stresses with those calculated from the Poiseuille equation yielded a difference of no more than 4 per cent in all the simulated conditions, despite the fact that the radial velocity can be 30 per cent larger than the axial velocity in certain cases. Poiseuille's law yielded a resonable estimation of the shear stresses; however, it remains to be validated in the case of a lymphangion with valves.

The treatment of the valves in these models is rather simplistic. Reddy *et al.* [55] assumed the resistance of the valves to be dependent only on the vessel diameter and imposed the condition that the flow rate is positive in all lymphangions. Essentially, these assumptions neglect the action (and possible interaction) of the valves. Macdonald *et al.* [45] assigned a timing of 20 ms, but with a constant resistance to flow for the one-dimensional model. Macdonald [64] also developed a computational fluid dynamics model of a valve, but the leaflet motion was imposed rather than calculated from the interaction with the flow. Bertram *et al.* [56] considered the valves to have a resistance which was a function of the pressure drop across the valve. However, the nature of this relationship in humans is unknown and data for other species are scarce as well.

Damage or removal of nodes increases the occurrence of lymphoedema when compared with vessel removal, thus indicating the importance of nodes in lymph flow [7]. Yet, none of these models includes the effect of lymph nodes on the propulsion of lymph. In fact, to the authors' knowledge, no modelling of the flow or mass exchange in the nodes has been attempted to date. Lymph nodes have smooth muscle in their walls and have been shown to contract with lower frequency than vessels. They are thought to act as reservoirs as well as pumps and filters. Their resistance to flow is quite high. Yet, there are a few studies of lymph node biomechanical behaviour; none of these includes lymph flow, but rather they concentrate on the contraction of the node's smooth muscle layer [29,81].

The computational models discussed to this point model the lymphatic system as a network of lymphangions; they are not concerned with the initial lymphatics. The first attempt to model the primary lymphatic valves should probably be credited to Reddy & Patel [42]. The flow was assumed to be axisymmetric and driven by a pressure difference between the interstitial space and the lymphatic vessel. The capillary was assumed to be supported by a linear elastic spring to account for the anchoring filaments. The authors added a hoop stress in the momentum equation of the vessel wall, even though the capacity of endothelial cells to produce a circumferential stress is not generally accepted [34]. Nevertheless, some interesting results were derived. It was found that, by increasing the amplitude of pressure pulses in the adjacent collecting vessel, the flow rate in the initial vessel increased; a result which is in line with the hypothesis that suction from collecting vessels facilitates lymph formation. Changes in interstitial fluid pressure also affected the filling of capillaries, which again is in agreement with the general concept of lymph formation. In general, though, the model suffered from lack of data regarding the initial lymphatics and therefore some parameters had to be estimated based on properties of blood vessels.

The optimal structure of the lymphatic capillary network was the focus of a study by Roose & Swartz [63]. Using homogenization theory, the authors developed a model that captures the influence of the microstructure of the capillary network on the macroscale fluid drainage at the tissue level. It was found that a hexagonal network is optimal in terms of fluid drainage, which is actually the structure found in mouse tail and human skin. It is interesting to note that the results obtained for the fluid clearance with respect to time at various positions along the network are qualitatively quite similar to those obtained by Reddy & Patel [42].

Mendoza & Schmid-Schonbein [61] developed a model of the initial lymphatics, but focused more on the endothelial cells that form the primary valves. The cells were modelled as elastic beams with the assumption of small deflections that were subjected to a fluid pressure difference generated by one-dimensional creeping flow into the lymphatics. It is interesting to note that, although 8 years had elapsed since the work by Reddy & Patel [42], the authors had to use data from vascular endothelial cells owing to the lack of data for lymphatics.

In the work by Mendoza & Schmid-Schonbein [61], small strains were assumed. The more elaborate model by Galie & Spilker [62] assumes large strains. This model also assumed two-dimensional flow in contrast to the model of Mendoza & Schmid-Schonbein [61]. The interstitial space was included as a porous medium, neglecting deformations and describing flow with Brinkman's equations. The model was completed by appropriate no-slip boundary conditions at the stationary walls, velocity matching at the fluid–structure interface, pressure and velocity continuity at the porous fluid interface and pressure at the boundaries. The authors concluded that these additions had an effect on the results as the calculated cell deflections were large. Both models still lack appropriate material properties for lymphatic cells.

## 5. Future work and conclusions

**Figure 5.**
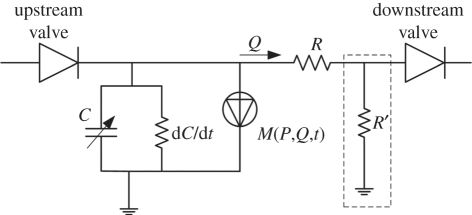
Model of a lymphangion.

The lymphatic system is a highly complex active system; a fact that when combined with the scarcity of anatomical and physiological data results in a formidable problem to model with mathematical methods, either analytically or numerically. In this section, a lumped model initially developed for the ventricle of the heart by Formaggia *et al.* [82] is proposed as an alternative model for a lymphangion. A lumped approach has the benefit of simplifying the problem, but at the same time yields a model that can be used to model large parts of or even the entire lymphatic circulation. The electrical analogue model proposed by Formaggia *et al.* [82] is shown in figure 5. Despite the fact that this was proposed for the heart, the resemblance of lymphangion function to that of a ventricle makes this model suitable as a starting point.

The lack of anatomical data favours the use of lumped models, as the spatial dimensions are reduced to zero. The lymphatic one-way valves are represented by diodes. The compliance of the wall is represented by a capacitor *C* and a time-varying resistance d*C*/d*t*. The action of the smooth muscle is modelled as a current source *M*(*P*,*Q*,*t*), which is a function of pressure *P*, flow rate *Q* and time *t*. The proposed model alleviates the limitations of the time-varying elastance concept used in some of the models discussed previously. It is also readily extensible. The addition of an extra resistor *R*′ connected to the ground (shown as the dashed line box in figure 5) allows for leakage of the vessel to be taken into account. The addition of an inductor in series with resistor *R* (not shown) can account for inertial effects of the fluid.

The parameters of the components can be easily altered for parametric studies, or can be made a function of other factors such as neural or hormonal stimulation. Unit models can be connected to form chains of lymphangions and bifurcations in order to model larger parts, or even the entire lymphatic circulation. The model can be connected with lumped models of the systemic circulation as well as in order to study the interaction of the two systems. More complicated multi-scale models can be constructed, in which parts of the system are modelled as lumped models and others as continuum one-dimensional (or higher) ones, as has been done for the cardiovascular system [83]. Interestingly, the same model, with appropriate component parameters, can be used to model the lymph nodes as well.

The paradigm for modelling of physiological flows has been set by the effort undertaken in the cardiovascular system. The abovementioned model is not the only one that can be drawn from the heavily researched systemic circulation. The methodologies developed for the systemic circulation range from simple Windkessel models [84] and multi-physics/multi-scale [85] to models of cardiac cells [86] and excitable tissues [87], and in principle may be applied to the lymphatic system as well.

The models of the lymphatic system discussed in this paper as well as the models of the cardiovascular system discussed in the previous paragraph are all based on continuum mechanics, but this is not the only possible approach to modelling biological systems; in fact, a continuum-based approach is not the most suitable for describing mechanobiological events [88]. Alternatives to phenomenological continuum mechanics models that can incorporate the activities of cells also exist. Cellular automaton and agent-based models of cellular growth and cell–cell interaction have existed for quite some time in the literature. These methods have also been coupled with continuum mechanics to study arterial adaptation [88,89]. Another approach is the theory of active particles [90–93]. It extends the kinetic theory of particles by assigning a state that defines the microscopic behaviour of each particle. Thus, macroscopic models of tissues can be derived based on the underlying behaviour of cells, cell–cell interaction, cell proliferation and death. These powerful mathematical tools afford the means to integrate events at the molecular, cellular and tissue levels, with the prospect of improved models which will find application in developmental biology, angiogenesis, lymphangiogenesis, tissue remodelling and tumour growth as well as interaction of cancer cells with those of the immune system.

In relation to the cardiovascular system, in which modelling efforts have been numerous and have advanced to the level of multi-scale and multi-physics even including models of excitable tissues, the work carried out on the lymphatic system seems to be in its infancy. More work will be necessary in combination with experimental verification in order to progress the knowledge on the function of the lymphatic system in health and disease, but, as our knowledge and understanding of the lymphatic system increases, so too will the likelihood that new treatments will emerge.

## Footnotes

1 An oblique layer has been reported by Telinius *et al*. [14] in the human thoracic duct.

2 Although smooth muscle cells are present in bovine valve leaflets, no evidence of an active valve mechanism has been reported in the literature [23].

3 Also called the lymphoid lobule [37].

4 These uncertainties resulted from the specific method employed by the authors, as reported by Macdonald *et al.* [45].

## References

[1] Charman W. N. Stella V. J. 1992 Lymphatic transport of drugs. Boca Raton, FL: CRC Press

[2] Swartz M. A. Hubbell J. A. Reddy S. T. 2008 Lymphatic drainage function and its immunological implications: from dendritic cell homing to vaccine design. Semin. Immunol. 20, 147–156 10.1016/j.smim.2007.11.007 (doi:10.1016/j.smim.2007.11.007) 18201895

[3] Swartz M. A. 2001 The physiology of the lymphatic system. Adv. Drug Deliv. Rev. 50, 3–20 10.1016/S0169-409X(01)00150-8 (doi:10.1016/S0169-409X(01)00150-8) 11489331

[4] Swartz M. A. Skobe M. 2001 Lymphatic function, lymphangiogenesis, and cancer metastasis. Microsc. Res. Tech. 55, 92–99 10.1002/jemt.1160 (doi:10.1002/jemt.1160) 11596154

[5] Dietrich T. Bock F. Yuen D. Hos D. Bachmann B. O. Zahn G. Wiegand S. Chen L. Cursiefen C. 2010 Cutting edge: lymphatic vessels, not blood vessels, primarily mediate immune rejections after transplantation. J. Immunol. 184, 535–539 10.4049/jimmunol.0903180 (doi:10.4049/jimmunol.0903180) 20018627 PMC4725297

[6] Tretbar L. L. Morgan L. C. Lee B. B. Simonian J. S. Bloneau B. 1992 Lymphedema: diagnosis and treatment. Berlin, Germany: Springer

[7] Földi M. Földi E. Kubik S. (eds) 2003 Textbook of lymphology for physicians and lymphedema therapists. Munich, Germany: Urban & Fischer

[8] Rockson S. G. 2010 Current concepts and future directions in the diagnosis and management of lymphatic vascular disease. Vasc. Med. 15, 223–231 10.1177/1358863X10364553 (doi:10.1177/1358863X10364553) 20483987

[9] Quere I. 2010 Lymphatic system: anatomy, histology and physiology. Presse Med. 39, 1269–1278 10.1016/j.lpm.2010.09.009 (doi:10.1016/j.lpm.2010.09.009) 21087839

[10] Leak L. V. Burke J. F. 1968 Ultrastructural studies on the lymphatic anchoring filaments. J. Cell Biol. 36, 129–149 10.1083/jcb.36.1.129 (doi:10.1083/jcb.36.1.129) PMC2107348 19806699

[11] Trzewik J. Mallipattu S. K. Artmann G. M. Delano F. A. Schmid-Schonbein G. W. 2001 Evidence for a second valve system in lymphatics: endothelial microvalves. FASEB J. 15, 1711–1717 10.1096/fj.01-0067com (doi:10.1096/fj.01-0067com) 11481218

[12] Sacchi G. Weber E. Agliano M. Raffaelli N. Comparini L. 1997 The structure of superficial lymphatics in the human thigh: precollectors. Anat. Rec. 247, 53–62 10.1002/(SICI)1097-0185(199701)247:1<53::AID-AR8>3.0.CO;2-G (doi:10.1002/(SICI)1097-0185(199701)247:1<53::AID-AR8>3.0.CO;2-G) 8986303

[13] Boggon R. P. Palfrey A. J. 1973 Microscopic anatomy of human lymphatic trunks. J. Anat. 114, 389–405 4736747 PMC1271452

[14] Telinius N. Drewsen N. Pilegaard H. Kold-Petersen H. de Leval M. Aalkjaer C. Hjortdal V. Boedtkjer D. B. 2010 Human thoracic duct *in vitro*: diameter-tension properties, spontaneous and evoked contractile activity. Am. J. Physiol. Heart Circ. Physiol. 299, H811–H818 10.1152/ajpheart.01089.2009 (doi:10.1152/ajpheart.01089.2009) 20511415

[15] Benoit J. N. Zawieja D. C. Goodman A. H. Granger H. J. 1989 Characterization of intact mesenteric lymphatic pump and its responsiveness to acute edemagenic stress. Am. J. Physiol. 257, H2059–H2069 2603989 10.1152/ajpheart.1989.257.6.H2059

[16] Nisimaru Y. 1982 Summary of our studies concerning the structure and function of lymphatic-system. Hiroshima J. Med. Sci. 31, 145–160 7174350

[17] Pan W. R. Le Roux C. M. Levy S. M. Briggs C. A. 2010 The morphology of the human lymphatic vessels in the head and neck. Clin. Anat. 23, 654–661 10.1002/ca.21004 (doi:10.1002/ca.21004) 20533512

[18] Pan W. R. le Roux C. M. Levy S. M. 2011 Alternative lymphatic drainage routes from the lateral heel to the inguinal lymph nodes: anatomic study and clinical implications. ANZ J. Surg. 81, 431–435 10.1111/j.1445-2197.2010.05639.x (doi:10.1111/j.1445-2197.2010.05639.x) 22295345

[19] Lee S. H. Wen H. J. Shen C. L. 1993 Ultrastructure of the monkey thoracic-duct and the cisterna chyli. J. Anat. 182, 205–212 8376195 PMC1259831

[20] Mazzoni M. C. Skalak T. C. Schmid-Schönbein G. W. 1987 Structure of lymphatic valves in the spinotrapezius muscle of the rat. Blood Vessels 24, 304–312 10.1159/000158707 (doi:10.1159/000158707) 3651619

[21] Gashev A. A. 2008 Lymphatic vessels: pressure- and flow-dependent regulatory reactions. In The lymphatic continuum revisited (ed. Rockson S. G.), pp. 100–109 Annals of the New York Academy of Sciences, vol. 1131. Oxford, UK: Blackwell Publishing

[22] Gashev A. A. Zawieja D. C. 2001 Physiology of human lymphatic contractility: a historical perspective. Lymphology 34, 124–134 11549124

[23] Davis M. J. Rahbar E. Gashev A. A. Zawieja D. C. Moore J. E. 2011 Determinants of valve gating in collecting lymphatic vessels from rat mesentery. Am. J. Physiol. Heart Circ. Physiol. 301, H48–H60 10.1152/ajpheart.00133.2011 (doi:10.1152/ajpheart.00133.2011) 21460194 PMC3129915

[24] Ohtani O. Ohtani Y. 2008 Structure and function of rat lymph nodes. Arch. Histol. Cytol. 71, 69–76 10.1679/aohc.71.69 (doi:10.1679/aohc.71.69) 18974599

[25] Roozendaal R. Mebius R. E. Kraal G. 2008 The conduit system of the lymph node. Int. Immunol. 20, 1483–1487 10.1093/intimm/dxn110 (doi:10.1093/intimm/dxn110) 18824503

[26] Hughes G. A. Allen J. M. 1993 Neural modulation of bovine mesenteric lymph-node contraction. Exp. Physiol. 78, 663–674 8240797 10.1113/expphysiol.1993.sp003714

[27] McGeown J. G. Gallagher M. J. P. 1990 The effects of field stimulation on bovine mesenteric lymph-node contractility. Pflugers Arch. Eur. J. Physiol. 416, 667–672 10.1007/BF00370613 (doi:10.1007/BF00370613) 1978934

[28] Thornbury K. D. McHale N. G. Allen J. M. Hughes G. 1990 Nerve-mediated contractions of sheep mesenteric lymph-node capsules. J. Physiol. Lond. 422, 513–522 1972193 10.1113/jphysiol.1990.sp017998 PMC1190146

[29] Tumer A. Ozturkdemir N. Basareroglu C. Noyan A. 1983 Spontaneous contractions and stretch-evoked responses of isolated lymph-nodes. J. Muscle Res. Cell Motil. 4, 103–113 10.1007/BF00711961 (doi:10.1007/BF00711961) 6841590

[30] McHale N. G. Roddie I. C. 1976 Effect of transmural pressure on pumping activity in isolated bovine lymphatic vessels. J. Physiol. Lond. 261, 255–269 988184 10.1113/jphysiol.1976.sp011557 PMC1309140

[31] McHale N. G. Meharg M. K. 1992 Coordination of pumping in isolated bovine lymphatic vessels. J. Physiol. Lond. 450, 503–512 1432715 10.1113/jphysiol.1992.sp019139 PMC1176134

[32] Ohhashi T. Azuma T. Sakaguchi M. 1980 Active and passive mechanical characteristics of bovine mesenteric lymphatics. Am. J. Physiol. 239, H88–H95 7396023 10.1152/ajpheart.1980.239.1.H88

[33] Zawieja D. C. Davis K. L. Schuster R. Hinds W. M. Granger H. J. 1993 Distribution, propagation, and coordination of contractile activity in lymphatics. Am. J. Physiol. 264, H1283–H1291 8476104 10.1152/ajpheart.1993.264.4.H1283

[34] Schmid-Schonbein G. W. 1990 Microlymphatics and lymph flow. Physiol. Rev. 70, 987–1028 2217560 10.1152/physrev.1990.70.4.987

[35] Browse N. L. Doig R. L. Sizeland D. 1984 The resistance of a lymph-node to lymph-flow. Br. J. Surg. 71, 192–196 10.1002/bjs.1800710308 (doi:10.1002/bjs.1800710308) 6697120

[36] Papp M. Makara G. B. Hajtman B. 1971 Resistance of *in situ* perfused lymph trunks and lymph nodes to flow. Experientia 27, 391–392 10.1007/BF02137266 (doi:10.1007/BF02137266) 5581094

[37] Willard-Mack C. L. 2006 Normal structure, function, and histology of lymph nodes. Toxicol. Pathol. 34, 409–424 10.1080/01926230600867727 (doi:10.1080/01926230600867727) 17067937

[38] Kowala M. C. Schoefl G. I. 1986 The popliteal lymph-node of the mouse: internal architecture, vascular distribution and lymphatic supply. J. Anat. 148, 25–46 3693091 PMC1261588

[39] Gretz J. E. Anderson A. O. Shaw S. 1997 Cords, channels, corridors and conduits: critical architectural elements facilitating cell interactions in the lymph node cortex. Immunol. Rev. 156, 11–24 10.1111/j.1600-065X.1997.tb00955.x (doi:10.1111/j.1600-065X.1997.tb00955.x) 9176696

[40] Aukland K. Reed R. K. 1993 Interstitial-lymphatic mechanisms in the control of extracellular fluid volume. Physiol. Rev. 73, 1–78 8419962 10.1152/physrev.1993.73.1.1

[41] Gashev A. A. Zawieja D. C. 2010 Hydrodynamic regulation of lymphatic transport and the impact of aging. Pathophysiology 17, 277–287 10.1016/j.pathophys.2009.09.002 (doi:10.1016/j.pathophys.2009.09.002) 20226639 PMC5507682

[42] Reddy N. P. Patel K. 1995 A mathematical-model of flow-through the terminal lymphatics. Med. Eng. Phys. 17, 134–140 10.1016/1350-4533(95)91885-K (doi:10.1016/1350-4533(95)91885-K) 7735643

[43] Schmid-Schonbein G. W. 1990 Mechanisms causing initial lymphatics to expand and compress to promote lymph flow. Arch. Histol. Cytol. 53, 107–114 10.1679/aohc.53.Suppl_107 (doi:10.1679/aohc.53.Suppl_107) 2252623

[44] Florey H. 1927 Observations on the contractility of lacteals: Part I. J. Physiol. 62, 267–272 16993848 10.1113/jphysiol.1927.sp002357 PMC1514841

[45] Macdonald A. J. Arkill K. P. Tabor G. R. McHale N. G. Winlove C. P. 2008 Modeling flow in collecting lymphatic vessels: one-dimensional flow through a series of contractile elements. Am. J. Physiol. Heart Circ. Physiol. 295, H305–H313 10.1152/ajpheart.00004.2008 (doi:10.1152/ajpheart.00004.2008) 18487438

[46] Muthuchamy M. Zawieja D. 2008 Molecular regulation of lymphatic contractility. In The lymphatic continuum revisited (ed. Rockson S. G.), pp. 89–99 Annals of the New York Academy of Sciences, vol. 1131. Oxford, UK: Blackwell Publishing. 10.1196/annals.1413.008 18519962

[47] Aukland K. 2005 Arnold Heller and the lymph pump. Acta Physiol. Scand. 185, 171–180 10.1111/j.1365-201X.2005.01470.x (doi:10.1111/j.1365-201X.2005.01470.x) 16218922

[48] Olszewski W. L. 2002 Contractility patterns of normal and pathologically changed human lymphatics. In The lymphatic continuum: lymphatic biology and disease (ed. Rockson S. G.), pp. 52–63 Annals of the New York Academy of Sciences, vol. 979. New York, NY: New York Academy of Sciences. 10.1111/j.1749-6632.2002.tb04867.x 12543716

[49] Olszewski W. L. Engeset A. 1980 Intrinsic contractility of pre-nodal lymph vessels and lymph-flow in human leg. Am. J. Physiol. 239, H775–H783 7446752 10.1152/ajpheart.1980.239.6.H775

[50] Negrini D. Ballard S. T. Benoit J. N. 1994 Contribution of lymphatic myogenic activity and respiratory movements to pleural lymph-flow. J. Appl. Physiol. 76, 2267–2274 7928846 10.1152/jappl.1994.76.6.2267

[51] Gashev A. A. Davis M. J. Zawieja D. C. 2002 Inhibition of the active lymph pump by flow in rat mesenteric lymphatics and thoracic duct. J. Physiol. Lond. 540, 1023–1037 10.1113/jphysiol.2001.016642 (doi:10.1113/jphysiol.2001.016642) 11986387 PMC2290276

[52] Reddy N. P. 1986 Lymph circulation: physiology, pharmacology, and biomechanics. Crit. Rev. Biomed. Eng. 14, 45–91 3524994

[53] Reddy N. P. (1974). A discrete model of the lymphatic system..

[54] Reymond P. Merenda F. Perren F. Rufenacht D. Stergiopulos N. 2009 Validation of a one-dimensional model of the systemic arterial tree. Am. J. Physiol. Heart Circ. Physiol. 297, H208–H222 10.1152/ajpheart.00037.2009 (doi:10.1152/ajpheart.00037.2009) 19429832

[55] Reddy N. P. Krouskop T. A. Newell P. H. 1977 Computer-model of lymphatic-system. Comput. Biol. Med. 7, 181–197 10.1016/0010-4825(77)90023-3 (doi:10.1016/0010-4825(77)90023-3) 891141

[56] Bertram C. D. Macaskill C. Moore J. E. 2011 Simulation of a chain of collapsible contracting lymphangions with progressive valve closure. J. Biomech. Eng. Trans. ASME 133, 011008 10.1115/1.4002799 (doi:10.1115/1.4002799) PMC3356777 21186898

[57] Quick C. M. Venugopal A. M. Dongaonkar R. M. Laine G. A. Stewart R. H. 2008 First-order approximation for the pressure–flow relationship of spontaneously contracting lymphangions. Am. J. Physiol. Heart Circ. Physiol. 294, H2144–H2149 10.1152/ajpheart.00781.2007 (doi:10.1152/ajpheart.00781.2007) 18326809

[58] Venugopal A. M. Stewart R. H. Laine G. A. Quick C. M. 2010 Nonlinear lymphangion pressure–volume relationship minimizes edema. Am. J. Physiol. Heart Circ. Physiol. 299, H876–H882 10.1152/ajpheart.00239.2009 (doi:10.1152/ajpheart.00239.2009) 20601461 PMC2944481

[59] Drake R. E. Allen S. J. Katz J. Gabel J. C. Laine G. A. 1986 Equivalent-circuit technique for lymph-flow studies. Am. J. Physiol. 251, 1090–1094 10.1152/ajpheart.1986.251.5.H1090 3777196

[60] Rahbar E. Moore J. E. J. 2011 A model of a radially expanding and contracting lymphangion. J. Biomech. 21, 118–123 10.1016/j.jbiomech.2011.02.018 PMC3086717 21377158

[61] Mendoza E. Schmid-Schonbein G. W. 2003 A model for mechanics of primary lymphatic valves. J. Biomech. Eng. Trans. ASME 125, 407–414 10.1115/1.1568128 (doi:10.1115/1.1568128) 12929246

[62] Galie P. Spilker R. L. 2009 A two-dimensional computational model of lymph transport across primary lymphatic valves. J. Biomech. Eng. Trans. ASME 131, 111004 10.1115/1.3212108 (doi:10.1115/1.3212108) 20353255

[63] Roose T. Swartz M. A. 2011 Multiscale modeling of lymphatic drainage from tissues using homogenization theory. J. Biomech. 45, 107–115 10.1016/j.jbiomech.2011.09.015 (doi:10.1016/j.jbiomech.2011.09.015) 22036032

[64] Macdonald A. J. (2008). The computational modelling of collecting lymphatic vessels..

[65] Drake R. E. Weiss D. Gabel J. C. 1991 Active lymphatic pumping and sheep lung lymph-flow. J. Appl. Physiol. 71, 99–103 1917771 10.1152/jappl.1991.71.1.99

[66] Eisenhoffer J. Elias R. M. Johnston M. G. 1993 Effect of outflow pressure on lymphatic pumping *in vitro*. Am. J. Physiol. 265, R97–R102 8342703 10.1152/ajpregu.1993.265.1.R97

[67] Rajagopalan S. Stewart R. H. Venugopal A. M. Laine G. A. Zawieja D. C. Quick C. M. 2003 Evaluating the time-varying elastance concept for lymphangions. In Proc. 25th Int. Conf. IEEE Engineering in Medicine and Biology Society, Cancun, Mexico, 17–21 September 2003. New York, NY: IEEE.

[68] Drake R. E. Dhother S. Oppenlander V. M. Gabel J. C. 1996 Lymphatic pump function curves in awake sheep. Am. J. Physiol. Regulatory Integr. Comp. Physiol. 270, R486–R488 10.1152/ajpregu.1996.270.2.R486 8779883

[69] Sunagawa K. Maughan W. L. Burkhoff D. Sagawa K. 1983 Left-ventricular interaction with arterial load studied in isolated canine ventricle. Am. J. Physiol. 245, H773–H780 6638199 10.1152/ajpheart.1983.245.5.H773

[70] Quick C. M. Venugopal A. M. Gashev A. A. Zawieja D. C. Stewart R. H. 2007 Intrinsic pump-conduit behavior of lymphangions. Am. J. Physiol. Regul. Integr. Comp. Physiol. 292, R1510–R1518 10.1152/ajpregu.00258.2006 (doi:10.1152/ajpregu.00258.2006) 17122333

[71] Venugopal A. M. Stewart R. H. Laine G. A. Dongaonkar R. M. Quick C. M. 2007 Lymphangion coordination minimally affects mean flow in lymphatic vessels. Am. J. Physiol. Heart Circ. Physiol. 293, H1183–H1189 10.1152/ajpheart.01340.2006 (doi:10.1152/ajpheart.01340.2006) 17468331

[72] Reddy N. P. Kesavan S. K. 1989 Low Reynolds-number liquid propulsion in contracting tubular segments connected through valves. Math. Comput. Modell. 12, 839–844 10.1016/0895-7177(89)90138-6 (doi:10.1016/0895-7177(89)90138-6)

[73] Danielsen M. Ottesen J. T. 2001 Describing the pumping heart as a pressure source. J. Theoret. Biol. 212, 71–81 10.1006/jtbi.2001.2348 (doi:10.1006/jtbi.2001.2348) 11527446

[74] Deserranno D. Kassemi M. Thomas J. D. 2007 Incorporation of myofilament activation mechanics into a lumped model of the human heart. Ann. Biomed. Eng. 35, 321–336 10.1007/s10439-006-9234-1 (doi:10.1007/s10439-006-9234-1) 17219084

[75] Reddy N. P. Krouskop T. A. Newell P. H. 1975 Biomechanics of a lymphatic vessel. Blood Vessels 12, 261–278 1182313 10.1159/000158062

[76] Hall J. G. Morris B. Woolley G. 1965 Intrinsic rhythmic propulsion of lymph in the unanaesthetized sheep. J. Physiol. 180, 336–349 5857113 10.1113/jphysiol.1965.sp007706 PMC1357389

[77] Dixon J. B. Zawieja D. C. Gashev A. A. Cote G. L. 2005 Measuring microlymphatic flow using fast video microscopy. J. Biomed. Opt. 10, 064016 10.1117/1.2135791 (doi:10.1117/1.2135791) 16409081

[78] Shapiro A. H. Jaffrin M. Y. Weinberg S. L. 1969 Peristaltic pumping with long wavelengths at low Reynolds number. J. Fluid Mech. 37, 799–825 10.1017/S0022112069000899 (doi:10.1017/S0022112069000899)

[79] Gashev A. A. 1991 [The mechanism of the formation of a reverse fluid filling in the lymphangions.] Fiziologicheskii Zhurnal SSSR Imeni I M Sechenova 77, 63–69 [In Russian.] 1668160

[80] Gravesen P. Branebjerg J. Jensen O. S. 1993 Microfluidics: a review. J. Micromech. Microeng. 3, 168 10.1088/0960-1317/3/4/002 (doi:10.1088/0960-1317/3/4/002)

[81] Lobov G. I. Pan'kova M. N. Dvoretsky D. P. Sergeev I. V. 2010 Characteristic of the active and passive mechanical properties of the lymph node capsule. Dokl. Biol. Sci. 434, 310–312 10.1134/S0012496610050054 (doi:10.1134/S0012496610050054) 20963651

[82] Formaggia L. Quarteroni A. Veneziani A. 2006 The circulatory system: from case studies to mathematical modeling. In Complex systems in biomedicine (eds Quarteroni A. Formaggia L. Veneziani A.), pp. 243–287 Milan, Italy: Springer

[83] Quarteroni A. Veneziani A. 2003 Analysis of a geometrical multiscale model based on the coupling of ODEs and PDEs for blood flow simulations. Multiscale Model. Simul. 1, 173–195 10.1137/S1540345902408482 (doi:10.1137/S1540345902408482)

[84] Westerhof N. Lankhaar J. W. Westerhof B. E. 2009 The arterial Windkessel. Med. Biol. Eng. Comput. 47, 131–141 10.1007/s11517-008-0359-2 (doi:10.1007/s11517-008-0359-2) 18543011

[85] Nordsletten D. A. Niederer S. A. Nash M. P. Hunter P. J. Smith N. P. 2011 Coupling multi-physics models to cardiac mechanics. Progr. Biophys. Mol. Biol. 104, 77–88 10.1016/j.pbiomolbio.2009.11.001 (doi:10.1016/j.pbiomolbio.2009.11.001) 19917304

[86] Fink M. 2011 Cardiac cell modelling: observations from the heart of the cardiac physiome project. Progr. Biophys. Mol. Biol. 104, 2–21 10.1016/j.pbiomolbio.2010.03.002 (doi:10.1016/j.pbiomolbio.2010.03.002) 20303361

[87] Nash M. P. Panfilov A. V. 2004 Electromechanical model of excitable tissue to study reentrant cardiac arrhythmias. Prog. Biophys. Mol. Biol. 85, 501–522 10.1016/j.pbiomolbio.2004.01.016 (doi:10.1016/j.pbiomolbio.2004.01.016) 15142759

[88] Hayenga H. N. Thorne B. C. Peirce S. M. Humphrey J. D. 2011 Ensuring congruency in multiscale modeling: towards linking agent based and continuum biomechanical models of arterial adaptation. Ann. Biomed. Eng. 39, 2669–2682 10.1007/s10439-011-0363-9 (doi:10.1007/s10439-011-0363-9) 21809144 PMC3207323

[89] Thorne B. C. Hayenga H. N. Humphrey J. D. Peirce S. M. 2011 Toward a multi-scale computational model of arterial adaptation in hypertension: verification of a multi-cell agent based model. Front. Physiol. 2, 20 10.3389/fphys.2011.00020 (doi:10.3389/fphys.2011.00020) 21720536 PMC3118494

[90] Bellomo N. Bellouquid A. Delitala M. 2008 From the mathematical kinetic theory of active particles to multiscale modelling of complex biological systems. Math. Comput. Modell. 47, 687–698 10.1016/j.mcm.2007.06.004 (doi:10.1016/j.mcm.2007.06.004)

[91] Bellomo N. Bellouquid A. Nieto J. Soler J. 2010 Complexity and mathematical tools toward the modelling of multicellular growing systems. Math. Comput. Modell. 51, 441–451 10.1016/j.mcm.2009.12.002 (doi:10.1016/j.mcm.2009.12.002)

[92] Bellomo N. Bianca C. Delitala M. 2009 Complexity analysis and mathematical tools towards the modelling of living systems. Phys. Life Rev. 6, 144–175 10.1016/j.plrev.2009.06.002 (doi:10.1016/j.plrev.2009.06.002) 20416852

[93] Bellouquid A. De Angelis E. 2011 From kinetic models of multicellular growing systems to macroscopic biological tissue models. Nonlinear Anal. Real World Appl. 12, 1111–1122 10.1016/j.nonrwa.2010.09.005 (doi:10.1016/j.nonrwa.2010.09.005)

